# Blocking P2X7 by intracerebroventricular injection of P2X7-specific nanobodies reduces stroke lesions

**DOI:** 10.1186/s12974-022-02601-z

**Published:** 2022-10-12

**Authors:** Maximilian Wilmes, Carolina Pinto Espinoza, Peter Ludewig, Joschi Stabernack, Arthur Liesz, Annette Nicke, Mathias Gelderblom, Christian Gerloff, Simonetta Falzoni, Eva Tolosa, Francesco Di Virgilio, Björn Rissiek, Nikolaus Plesnilla, Friedrich Koch-Nolte, Tim Magnus

**Affiliations:** 1 Department of Neurology, University Medical Center Hamburg-Eppendorf, Martinistraße 52, 20246 Hamburg, Germany; 2 Institute of Immunology, University Medical Center Hamburg-Eppendorf, Martinistraße 52, 20246 Hamburg, Germany; 3 Institute for Stroke and Dementia Research, University Medical Center, 813777 Munich, Germany; 4 SyNergy Cluster for Systems Neurology, 81377 Munich, Germany; 5 Walther Straub Institute of Pharmacology and Toxicology, Ludwig-Maximilians-Universität München, Munich, Germany; 6 Department of Medical Sciences, University of Ferrara, Ferrara, Italy

**Keywords:** P2X7, Nanobodies, Ischemic stroke, Nanomedicine, Cerebrovascular disease

## Abstract

**Background:**

Previous studies have demonstrated that purinergic receptors could be therapeutic targets to modulate the inflammatory response in multiple models of brain diseases. However, tools for the selective and efficient targeting of these receptors are lacking. The development of new P2X7-specific nanobodies (nbs) has enabled us to effectively block the P2X7 channel.

**Methods:**

Temporary middle cerebral artery occlusion (tMCAO) in wild-type (wt) and P2X7 transgenic (tg) mice was used to model ischemic stroke. Adenosine triphosphate (ATP) release was assessed in transgenic ATP sensor mice. Stroke size was measured after P2X7-specific nbs were injected intravenously (iv) and intracerebroventricularly (icv) directly before tMCAO surgery. In vitro cultured microglia were used to investigate calcium influx, pore formation via 4,6-diamidino-2-phenylindole (DAPI) uptake, caspase 1 activation and interleukin (IL)-1β release after incubation with the P2X7-specific nbs.

**Results:**

Transgenic ATP sensor mice showed an increase in ATP release in the ischemic hemisphere compared to the contralateral hemisphere or the sham-treated mice up to 24 h after stroke. P2X7-overexpressing mice had a significantly greater stroke size 24 h after tMCAO surgery. In vitro experiments with primary microglial cells demonstrated that P2X7-specific nbs could inhibit ATP-triggered calcium influx and the formation of membrane pores, as measured by Fluo4 fluorescence or DAPI uptake. In microglia, we found lower caspase 1 activity and subsequently lower IL-1β release after P2X7-specific nb treatment. The intravenous injection of P2X7-specific nbs compared to isotype controls before tMCAO surgery did not result in a smaller stroke size. As demonstrated by fluorescence-activated cell sorting (FACS), after stroke, iv injected nbs bound to brain-infiltrated macrophages but not to brain resident microglia, indicating insufficient crossing of the blood–brain barrier of the nbs. Therefore, we directly icv injected the P2X7-specific nbs or the isotype nbs. After icv injection of 30 µg of P2X7 specific nbs, P2X7 specific nbs bound sufficiently to microglia and reduced stroke size.

**Conclusion:**

Mechanistically, we can show that there is a substantial increase of ATP locally after stroke and that blockage of the ATP receptor P2X7 by icv injected P2X7-specific nbs can reduce ischemic tissue damage.

**Supplementary Information:**

The online version contains supplementary material available at 10.1186/s12974-022-02601-z.

## Introduction

Stroke induces sterile inflammation, which worsens the initial brain damage and neurological outcome [1, 2]. Hypoxic brain tissue releases many molecules, which can activate cells such as microglia in the surrounding tissue and lead to infiltration of other immune cells such as neutrophils, amplifying the inflammatory cascade [3]. These molecules include adenosine triphosphate (ATP) as well as nicotinamide adenine dinucleotide (NAD), heat shock protein (HSP), and high-mobility group box 1 protein (HMGB1). These factors can activate the inflammasome and induce the secretion of proinflammatory cytokines by innate immune cells [4, 5]. These molecules activate several pathways, such as the ATP/P2X7 pathway or the nuclear factor kappa-light-chain-enhancer of activated B cells (NFκB) pathway [6]. The P2X7 receptor is a homotrimeric, ligand-gated nonselective cation channel that is expressed in the central nervous system as well as on immune cells [7]. The P2X7 receptor consists of three polypeptide subunits, each with two transmembrane domains [8, 9]. After activation by extracellular ATP (eATP), these subunits form an ion-permeable channel, which induces Na^+^ and Ca^2+^ influx and K^+^ efflux, resulting in plasma membrane depolarization and initiation of Ca^2+^ signaling cascades. The K^+^ efflux through the P2X7 receptor supports the formation of the Nod-like receptor protein 3 (NLRP3)-mediated inflammasome complex, which cleaves pro-caspase 1 and leads to a subsequent cleavage of pro-IL-1β and pro-IL-18 into their biologically active forms [5, 10, 11]. The amount of accessible intracellular pro-IL-1β and pro-IL-18 also depends on another signal transmitted by receptors, such as Toll-like receptors (TLRs) or tumor necrosis factor (TNF)-receptors, and subsequent NFκB activation.

In the central nervous system (CNS), P2X7 has been found primarily on microglia, with less on astrocytes and oligodendrocytes [12–17]. These findings were confirmed by data from the Allen Brain Atlas for mice [18] and humans [19]. There are some similarities between human and rodent P2X7 expression in the brain, such as high expression on microglia and low expression on astrocytes, but there are also some differences such as high expression of P2X7 on human oligodendrocytes and low expression on rodent oligodendrocytes.

Several studies have shown that the experimental stroke size in P2X7^−/−^ mice is smaller than that in wild-type mice [20, 21]. In addition, blocking the P2X7 channel with brilliant blue G (BBG) attenuated ischemic damage [20]. However, systemic BBG cannot be used in humans since it is nonspecific and toxic.

Nanobodies (nbs), named for their small size (2.5 nm diameter, 4 nm height, 12 kDa) [22], are single-domain antibodies derived from camelid heavy chain antibodies. Compared to small molecule inhibitors, nbs have key advantages, such as low toxicity, high specificity, no off-target effects and, in the case of P2X7, a more potent inhibition [10, 21, 23]. With their long complementarity determining region 3 (CDR3), these molecules can access cavities or clefts on membrane proteins that are often inaccessible to antibodies [24, 25]. Other advantages of nbs over conventional antibodies include high stability, better solubility and rapid and targetable in vivo biodistribution. In addition, the ability to form nb multimers and the low costs and ease of production make them ideal candidates for treatment [26]. Fusion of an nb (monomer or multimer) to the Fc domain of a conventional antibody yields a heavy chain antibody with reconstituted Fc-mediated effector functions, including binding to Fc receptors, extended half-life and complement activation. This phenomenon allows a much broader tailoring of nbs than of conventional antibodies to different pathophysiologies [27].

In this proof-of-concept study, we used P2X7-specific nbs to treat mice directly before temporary middle cerebral artery occlusion (tMCAO) surgery. We found that these nbs need to be injected intracerebroventricularly to reach P2X7 receptor on brain resident cells and protect against ischemic stroke.

## Methods

### Animals

All animal experiments were approved by the local animal care committees (Behörde für Justiz und Veterinärwesen Hamburg, Nr 006/18) and conducted following the “Guide of the Care and Use of Laboratory Animals” published by the US National Institutes of Health (NIH Publication No. 83–123, revised 1996). All mice were kept at a constant temperature of 22 ± 2 °C with a 12-h light–dark cycle and ad libitum access to food and water. Only 12- to 18-week-old male mice were used for this study. C57BL/6J mice were purchased from Charles River (Bar Harbor, ME 04609, USA), whereas the generation of pmeLUC transgenic and P2X7-EGFP transgenic mice (line 17 in C57BL/6J) was described previously [28, 29].

### Production of P2X7 nbs

The P2X7-antagonizing nbs 1c81 and 13A7 were selected and cloned into the pCSE2.5 expression vector (kindly provided by Thomas Schirrmann, Braunschweig, Germany) [30] as described previously [10, 28]. Then, 13A7 was fused to the hinge, constant domain heavy chain (CH) 2, and CH3 domains of mouse immunoglobulin (Ig) G2c, resulting in a heavy chain format (nb A), whereas 1c81 was dimerized and fused to the albumin-specific nb Alb8 (mAb77) [31], resulting in a bispecific heterotrimeric nb with an extended half-life (nb B) (Additional file 1: Fig. S1). Since dimers showed a higher potency than monomers [10], we used nb B for intracerebroventricular (icv) injection. For icv injection, we needed to create a construct that could be highly concentrated without aggregation, so we modified our nb B (Additional file 1: Fig. S1). The modified nb B-mod was concentrated up to 15 μg/μl without aggregation. The exact sequences and further information on the various constructs can be found in patent WO/2013/178783.

HEK-6E cells were transfected with the constructs, and 6 days after transfection, the nbs were purified from the cell supernatant by affinity chromatography on a protein-G Sepharose column. The buffer was exchanged by gel filtration on a PD-10 column. The concentration and purity were monitored by sodium dodecyl sulfate–polyacrylamide gel electrophoresis (SDS-PAGE) and a BCA™ Protein Assay Kit (Pierce).

### tMCAO surgery and stroke size analysis

tMCAO was performed as previously described [32–34]. Mice were anesthetized with 1.5% isoflurane in 100% O_2_ and an intraperitoneal injection of 0.05 mg/kg body weight buprenorphine in saline was used as analgesic. A midline skin incision in the neck was made before ligating the proximal common carotid artery (CCA) and the external carotid artery (ECA) without disrupting the venous vessels. Vital parameters were continuously monitored with PhysioSuite (Kent Scientific Corporation, USA). Occlusion was confirmed by a laser Doppler monitor (moorVMS-LDF; Moor Instruments, UK) and persisted for 40 min. Mice with an occlusion rate of less than 80% were excluded.

Stroke size was measured by triphenyl tetrazolium chloride (TTC) staining and magnetic resonance imaging (MRI). We used a 7-Tesla MR small animal imaging system (ClinScan, Bruker, Ettlingen, Germany). The imaging protocol comprised T2-weighted imaging MRI. Calculation of corrected stroke volumes was performed as described previously [35].

The infarct volumes and total areas of the treated hemisphere were calculated using NIH ImageJ software.

### Intravenous (iv) and icv injections of nbs

Different methods of nb administration were used. P2X7-specific nbs (nb A, 13A7-Fc) were directly injected (100 µg in 100 µl of phosphate-buffered saline [PBS]) intravenously, or P2X7-specific nbs (nb B/nb B-mod, 1c81-dim-HLE) or isotype nbs against human cluster-of-differentiation (CD) 38 were injected (30 µg in 2 µl of PBS containing 60 mg/ml trehalose and 0.4 mg/ml Tween-20) directly into the ventricles of the brain by using a stereotaxic apparatus. Mice were pain treated with 1 mg tramadol/kg body weight one day before surgery. Directly before the surgery, the mice were anesthetized with isoflurane (4% for induction, 2.5% for maintenance) in 100% oxygen_._ After placing the mice in a stereotactic frame (Stoelting, 51615), we made a 1-cm-long incision above the midline. A cranial burr hole (0.9 mm) was drilled 1.1 mm lateral and 0.5 mm posterior to the bregma. Nbs were drawn into a 10-μl Hamilton syringe (Hamilton, 1701RN) connected to a 26-gauge needle (Hamilton, 26G, Point Style 4, 12°) controlled by a motorized stereotaxic injector (Stoelting, integrated stereotaxic injector [ISI]).

The needle was slowly introduced 2.3 mm deep into the left ventricle (Additional file 1: Fig. S2). Following a period of 5 min to let the ventricular system re-expand, 2 μl of dissolved nbs at a concentration of 15 μg/μl was injected at 1 μl/min. This step was followed by another 10-min break and slow removal of the needle. Vital parameters were monitored by an animal support unit (Minerve, Esternay, France). Body temperature was maintained throughout the procedure at 37 °C using a feedback-controlled heating device.

### In vivo ATP measurement after tMCAO using pmeLUC-TG

Three hours before tMCAO surgery, 150 mg/kg luciferin (Promega) was injected intraperitoneally. Luciferin was reinjected 1 day after tMCAO in prior of the measurement. In vivo ATP release was monitored by whole-body luminometry performed using the IVIS-Perkin Elmer in vivo imaging system. In vitro calibration was performed in brain homogenates from pmeLUC-tg mice.

### Microglia and macrophage preparation and FACS

Animals were euthanized and perfused with PBS. Brains were dissected and digested in 1 mg/ml collagenase A (Roche) and 0.1 mg/ml DNase type I (Sigma). Separation from myelin and debris was performed by density centrifugation with Percoll (GE Healthcare). The following antibodies and detection systems were used: CD45-APC-Cy7 (1:100, 30-F11, #103,115 BioLegend), CD45-PerCP (1:100, 30-F11, #103,129, BioLegend), CD11b-APC (1:100, M1/70, #17–0112-82, eBioscience), Ly6C-PerCP/Cy5.5 (1:100, HK1.4, #128,011, BioLegend), anti-mIgG1-brilliant violet (BV) 421 (1:100, RMG1-1, #406,615, BioLegend), anti-mIgG2-BV421 (1:100, RM223, #31–1103-02, Dianova), steptavidin-BV421 (1:100, #405,226, BioLegend), Fc blocking anti-CD16/CD32 (1:100, 2.4G2, #BE0307, BioXcell), and mAb77 (1:100, Alb8-specific mouse monoclonal antibody kindly provided by Ablynx). Microglia were gated as mentioned in the supplementary materials (Additional file 1: Fig. S3). In the first step, cells were incubated (30 min on ice) with Fc blocking anti-CD16/32, where ex vivo samples were incubated with 0.5 μg of P2X7 specific nb in the presence of Fc blocking anti-CD16/32. For detection of cell-bound P2X7 nbs, cells were incubated either with biotinylated anti-mouse IgG2c-fused antibody followed by streptavidin BV421 conjugated (nb A) or with mAb77 (nb B-mod) followed by fluorochrome-conjugated antibodies in the presence of Fc blocking anti-CD16/CD32 (Additional file 1: Fig. S1). Calcium influx was measured by a Fam-fluorochrome-labeled inhibitor of caspase-1 (FLICA) detection system. DAPI uptake and IL-1β release were monitored by flow cytometry. IL-1β enzyme-linked immunoassays (ELISAs) were performed according to Invitrogen Thermo Fisher Scientific (#BMS6002).

Differentiation between brain resident microglia and brain infiltrating macrophages was performed by FACS, where infiltrating cells were labeled CD45^+^CD11b^+^Ly6C^high^ and microglia were labeled CD45^int^CD11b^+^ [36].

For functional analysis, brain cells from icv injected brain cells were stimulated with 0.5 mM ATP in RPMI containing DAPI at 37 °C for 5 min. Cells were washed and analyzed by flow cytometry.

### Immunostaining

Mice were deeply anesthetized, and brains were fixed with 4% paraformaldehyde (PFA) by transcardial perfusion. After fixation in 4% PFA overnight, 50 µm thick sections were prepared using a vibratome. Immunostaining was performed at 4 °C on free-floating sections using an anti-Iba1 antibody to detect microglia (Fujifilm Wako Pure Chemical Corporation) and an anti-neuronal nuclear protein (NeuN) antibody to detect neurons (Thermo Fisher Scientific). DAPI (Thermo Fisher Scientific) was used to counterstain nuclei. Images were obtained by confocal laser scanning microscopy (LSM 880, Zeiss, Oberkochen, Germany).

## Results

### ATP is released rapidly after ischemic stroke

We analyzed ATP release after tMCAO by using ATP-sensing pmeLUC transgenic mice. These mice ubiquitously express firefly-derived luciferase on the outer layer of the plasma membrane [29, 37], which is activated by extracellular ATP. The pmeLUC mice can be used to detect changes in the extracellular ATP concentration in the micromolar range in a strictly ATP-selective fashion since the luciferase used is insensitive to all other nucleotides [37].

**Fig. 1:**
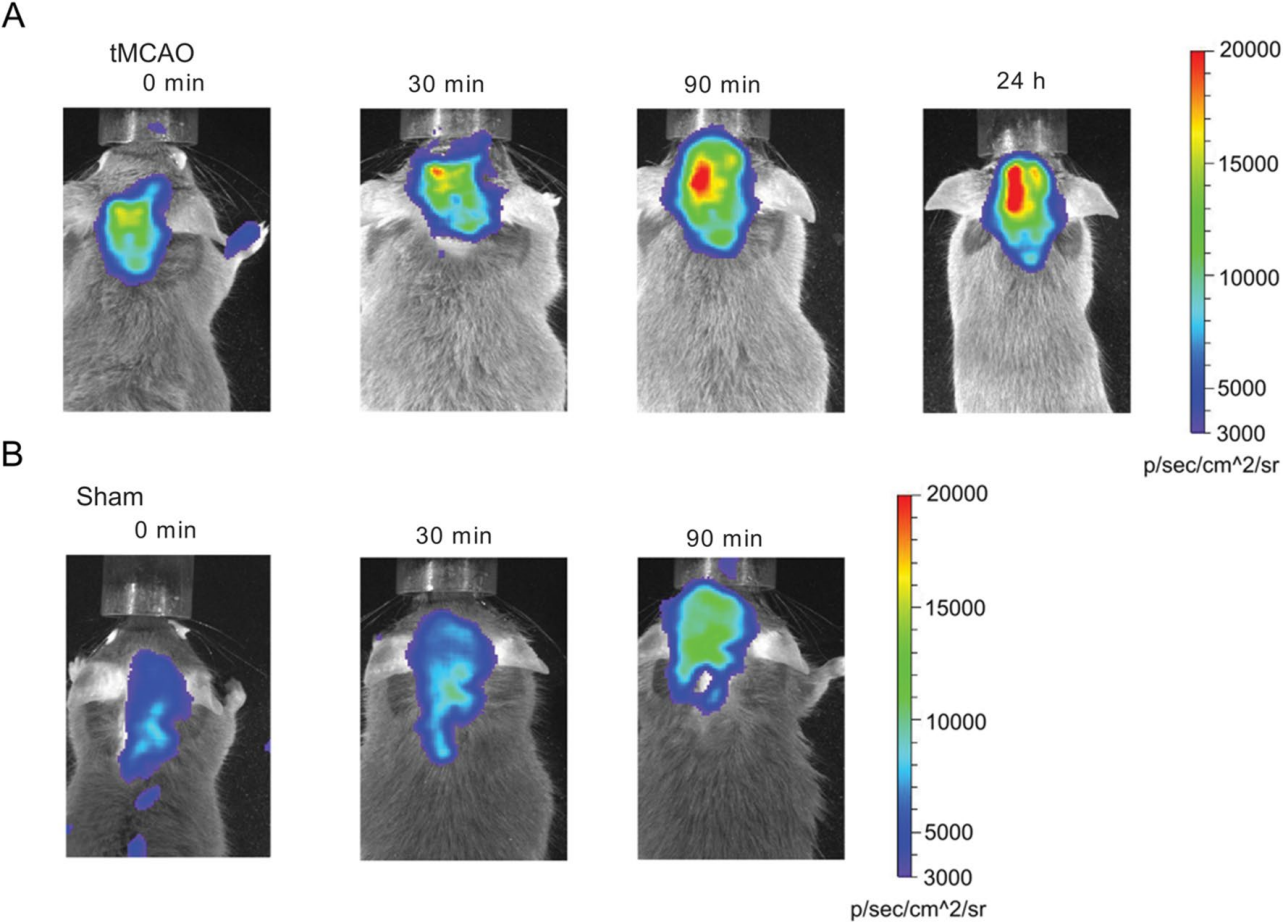
ATP was released after stroke. Transgenic ATP sensor mice (pmeLuc-mice) show eATP in the brain. **A** After tMCAO, ATP is rapidly released in the ischemic hemisphere starting after 30 min and is still prominent after 24 h compared to that in the contralateral hemisphere. **B** Sham-treated mice do not show substantial ATP level differences between the two hemispheres. Three mice for each group underwent surgery with similar results. Representative pictures are provided

Immediately after tMCAO, a base image was taken (Fig. 1). At 90 min after artery occlusion, eATP release/luminescence increased in the ischemic hemisphere. After 24 h, we could still detect a strong signal in the ischemic hemisphere. Rough estimations of the in vivo eATP concentration were performed by an in vitro concentration gradient (Additional file 1: Fig. S4).

### P2X7 overexpression exacerbates stroke volume

**Fig. 2:**
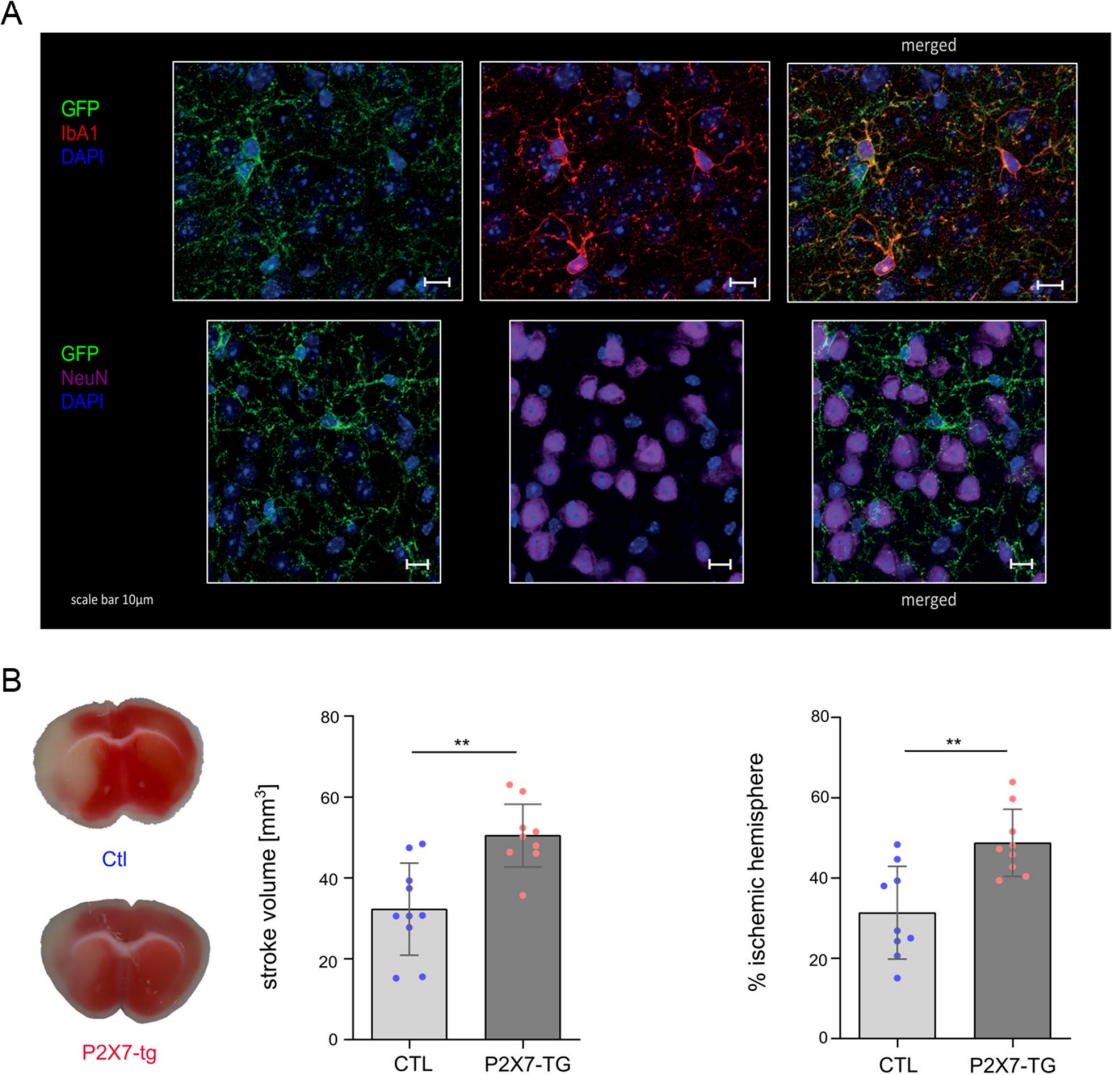
P2X7 was mainly expressed on microglia, and P2X7-overexpressing transgenic mice had more strokes. Immunostaining of 50 μm free-floating sections of P2X7-EGFP (**A**, *n* = 2) transgenic mice illustrated that P2X7 is highly expressed on IbA1-positive (red) cells compared to NeuN-positive (purple) neurons. DAPI (blue) was used to stain the nucleus. For the negative control, see Additional file 1: Fig. S5. **B** One day after tMCAO surgery, including an occlusion time of 40 min, the P2X7-overexpressing mice had a significantly higher infarct volume and a significantly higher % loss of parenchyma in the ischemic hemisphere. Statistical significance was analyzed by Student’s *t* test. **p < 0.001. Data are presented as the median ± range of 9 P2X7-overexpressing mice and 10 wild-type littermate controls. Representative TTC staining and representative overlays of stroke volume at day 1 following tMCAO treatment

Immunostaining of P2X7-enhanced green fluorescent protein (EGFP) transgenic mice revealed that P2X7 is expressed mainly on ionized calcium-binding adapter molecule 1 (IbA1)-positive cells (Fig. 2A, negative controls in Additional file 1: Fig. S5). Merged staining of IbA1 and GFP showed, for the most part, a congruent symmetry, where neurons stained with NeuN did not show any GFP expression.

Additionally, we used the expression data from the Allen Brain Atlas for mice [18] and humans [19] to determine cellular P2X7 expression in the brain (Table 1).

**Table 1:** Expression of P2X receptor genes in brain resident cells in humans and mice

Cell class	Species	P2X1R	P2X2R	P2X3R	P2X4R	P2X5R	P2X6R	P2X7R	Panx1
All	Mice	0.332	0.511	1.216	19.836	1.051	3.929	7.355	29.659
	Human	0.292	0.339	0.445	10.320	5.952	6.170	41.939	13.923
Non-Neuronal	Mice	2.397	0.000	1.514	46.330	0.524	7.071	80.562	6.860
	Human	2.705	0.003	0.641	15.760	0.002	1.908	120.507	8.173
Endothelial	Mice	0.003	0.000	0.116	17.535	0.563	0.000	5.314	3.262
	Human	45.215	0.000	0.000	3.565	0.000	0.000	0.067	2.641
Microglia/PVM	Mice	13.870	0.000	0.067	132.048	0.000	0.617	390.551	12.421
	Human	26.250	0.000	0.074	92.659	0.000	0.054	226.445	16.611
Astrocytes	Mice	0.012	0.000	0.898	28.560	0.327	10.739	17.652	0.044
	Human	0.176	0.000	1.042	11.586	0.002	0.507	26.116	1.883
Oligo- dendrocytes	Mice	0.000	0.000	2.622	46.636	0.000	0.359	22.786	11.473
	Human	1.076	0.009	0.812	12.157	0.003	2.621	189.904	7.268
OPC	Mice	5.271	0.000	9.382	20.593	5.435	0.000	122.913	56.927
	Human	0.098	0.000	0.098	5.707	0.000	3.245	121.165	15.029
Excitatory Neurons	Mice	0.369	0.008	1.331	23.817	0.993	5.603	4.603	25.911
	Human	0.149	0.480	0.452	10.647	6.711	5.875	45.346	11.203
Inhibitory Neurons	Mice	0.117	1.118	1.084	13.174	1.170	1.884	4.010	36.270
	Human	0.119	0.052	0.383	8.268	5.328	7.877	15.680	22.188

Expression data from the Allen Brain Atlas for mice [18] and humans [19] on P2X receptors and pannexin 1. P2X7 expression is mainly found on human and rodent microglia and perivascular macrophages (PVM). Data are given as the mean count per million (CPM). Other cells show substantially lower expression of P2X7. Although oligodendrocytes show high expression of P2X7 in humans, this cannot be transferred to rodents. Oligo precursor cells (OPC) show intermediate P2X7 expression in humans and rodents

To evaluate the relevance of P2X7 for ischemic stroke, we used P2X7-overexpressing mice [28]. Littermate mice (*n* = 10) and P2X7-overexpressing mice (*n* = 9) were subjected to tMCAO, and the stroke size was determined by TTC staining. After 40 min of occlusion, we found that stroke sizes in P2X7-overexpressing mice was significantly larger in comparison to wt controls 24 h after tMCAO. The P2X7-overexpressing mice had a mean ischemic volume of 52.50 mm^3^ ± 8.52 mm^3^ compared to the littermates with a mean ischemic volume of 36.66 mm^3^ ± 13.64 mm^3^(Fig. 2B). Additionally, the percentage of the ischemic hemisphere differed significantly between the two cohorts, with 49.36% ± 8.76% in the P2X7-overexpressing cohort and 34.15% ± 13.56% in the control group (Fig. 2B).

### *P2X7*-specific nbs inhibit the P2X7 receptor on microglia in vitro

**Fig. 3:**
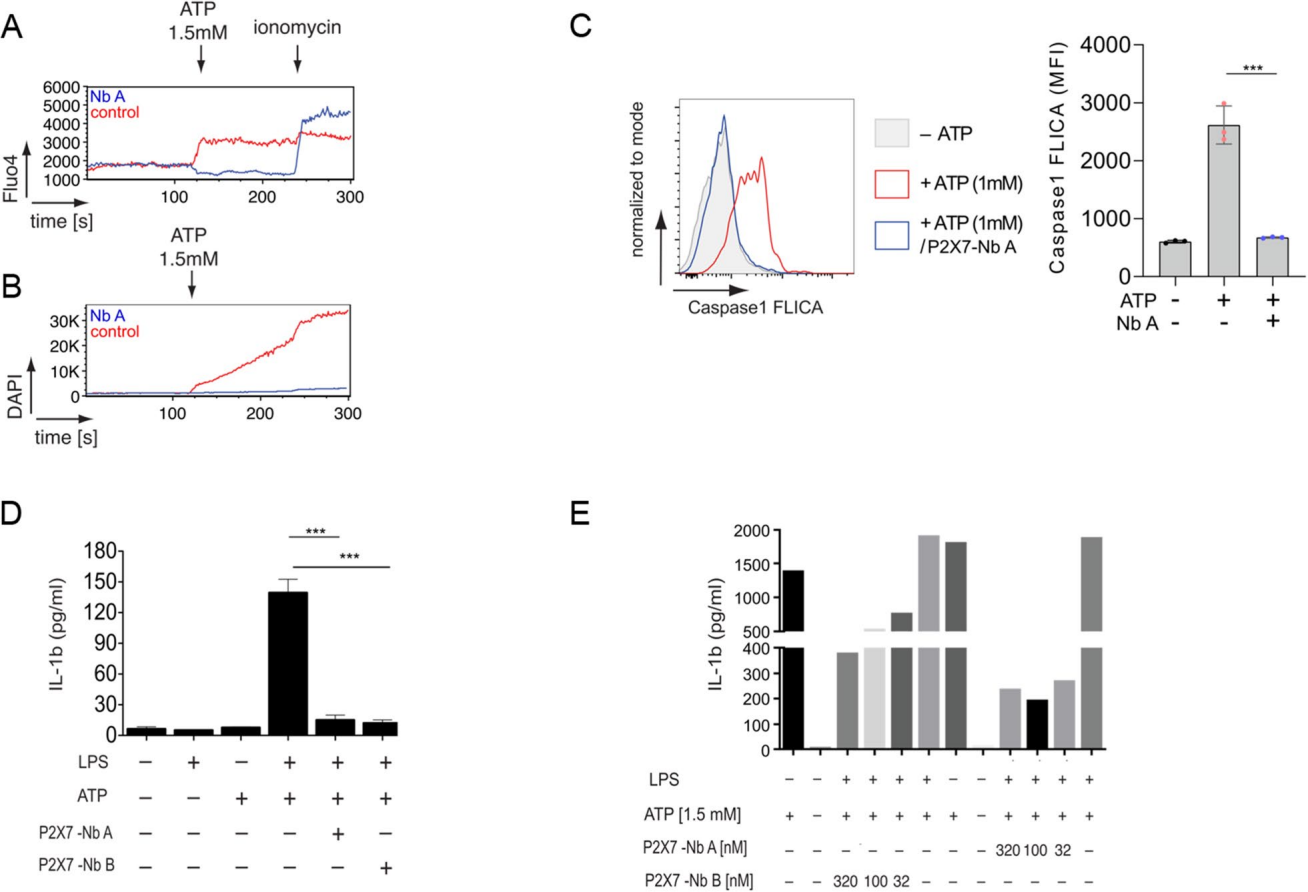
P2X7-specific nbs influenced the P2X7 pathway in vitro. Primary microglia preincubated with P2X7-specific nbs showed a substantially lower calcium influx (monitored by Fluo4 [**A**]) and DAPI uptake (**B**) than those of the isotype control group after ATP (1.5 mM) challenge. As a positive control, ionomycin facilitated calcium influx across the plasma membrane. **C** In the absence of P2X7-specific nbs, stimulated microglia showed significantly higher caspase 1 activation (measured by the appearance of FAM-FLICA; *n* = 3) than ATP-activated microglia in the presence of these nbs. **D** Blockade of P2X7 by specific nbs dampened IL-1β release in cultured microglia after ATP and LPS stimulation. Both types of P2X7-specific nbs used in this investigation significantly decreased IL-1β release (*n* = 3). The findings in **D** are specified in **E**, showing that IL-1β release can be reduced by using even low doses of nbs. Statistical significance was determined by Student’s t test. ***p < 0.0001

To verify that P2X7-specific nbs can inhibit the P2X7 receptor in microglia, we tested them in ATP-stimulated primary microglia in vitro. The P2X7-specific nbs (1 μg/ml) decreased the ATP-evoked calcium influx compared to the isotype control nbs (Fig. 3A). In addition, the P2X7-specific nbs dampened the ATP-evoked pore formation monitored by DAPI uptake (Fig. 3B). Caspase-1 activation, measured by the FAM-FLICA detection system, was reduced significantly in the presence of the P2X7-blocking nbs (Fig. 3C; mean fluorescence intensity [MFI]: 2617 vs. 674; ***p < 0.001; *n* = 3). LPS/ATP-evoked IL-1β release was significantly reduced after preincubation with the P2X7-specific nbs (Fig. 3D; ***p < 0.001; *n* = 3). Further investigation showed that low doses of nbs were sufficient to suppress IL-1β release (Fig. 3E).

### Iv injection of P2X7-specific nbs does not affect stroke size

**Fig. 4:**
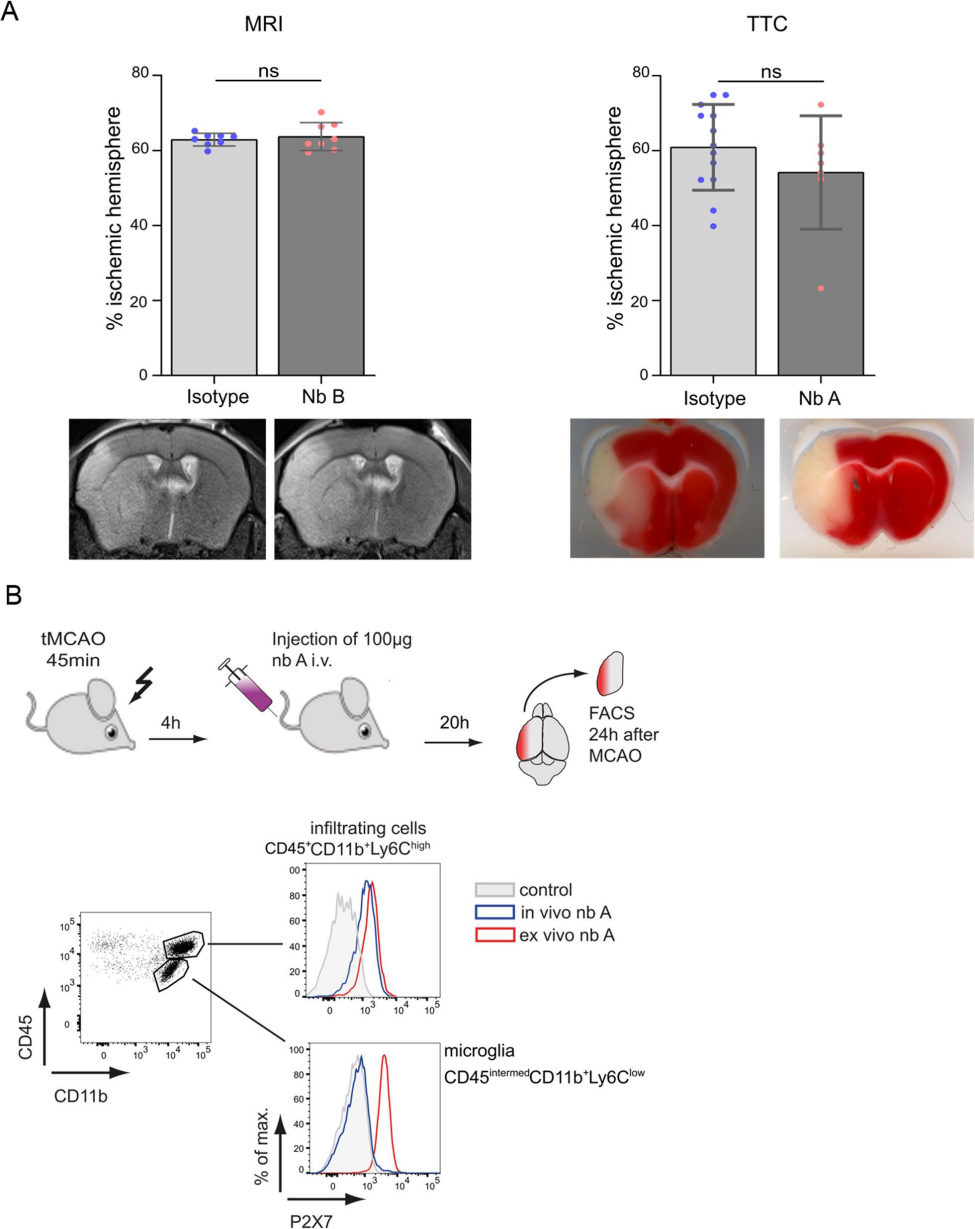
Intravenously injected P2X7-blocking nbs did not cross the BBB and did not influence stroke size. C57BL6J mice intravenously received 100 μg of P2X7-specific nbs or isotype control nbs after tMCAO surgery. Twenty-four hours after stroke, lesion size was measured via MRI and TTC. Between both groups, the % of parenchymal loss in the ischemic hemisphere did not differ significantly (**A**; TTC: isotype *n* = 13 vs. P2X7-specific nb *n* = 7; MRI: isotype *n* = 8 vs. P2X7-specific nb *n* = 8). Statistical significance was determine by Student’s t test. **B** For determination of whether nbs cross the BBB, the brains of iv nb-treated mice were analyzed 24 h after treatment and stroke. After injection of 100 μg of nbs conjugated with AlexaFluor647, infiltrating macrophages (C; CD45^+^CD11b^+^Ly6C^high^) were covered with P2X7-specific nbs, whereas brain resident microglia (C; CD45^+^CD11^intermed^Ly6C^low^) did not show any P2X7-specific nb on their surface by flow cytometry. As a positive control, 0.5 μg of nb A was added ex vivo

Next, we investigated the effect of the P2X7-specific nbs on stroke size in wild-type mice. We intravenously injected 100 μg of nb B prior to tMCAO. Stroke size was analyzed 24 h after surgery in two independent cohorts by histology or MRI. We did not find any significant reduction in stroke size compared to that of the isotype control nb group (MRI and TTC: *p* > 0.05). The isotype-treated wild-type mice showed ischemic lesions of 60.91% ± 12.23% in the ischemic hemisphere in the TTC cohort and 62.92% ± 1.68% in the MRI cohort. The nb-treated mice showed an almost identical ischemic lesions, with 59.41% ± 15.13% in the TTC cohort and 63.72% ± 3.73% in the MRI cohort (Fig. 4A).

To detect whether the nbs successfully cross the blood–brain barrier (BBB), we intravenously injected fluorophore-labeled P2X7-specific nbs 1 h after tMCAO. After 24 h, we analyzed the MFI of P2X7-specific nbs bound to either brain resident microglia or brain infiltrating macrophages (see staining protocol in Additional file 1: Fig. S1). We found that 100 µg of intravenously injected nbs did not label brain resident microglia but could be detected on infiltrating macrophages (Fig. 4B), indicating an insufficient passage through the BBB of the nbs. This insufficient passage was further verified by a dose–response analysis of intravenously injected nbs (Additional file 1: Fig. S6), indicating that extremely high doses are needed for BBB crossing.

### Icv injection of P2X7-specific nbs reduces stroke size

To circumvent the BBB, we performed direct icv injection of nbs into the brain and examined the effect of these P2X7-specific nbs on stroke size. Because of the minimal volume of 1–2 μl available for icv injections, we needed to concentrate the nbs. For this, we had to slightly modify nb B (see MM). After performing a dose–response analysis of icv injected nbs, we saw that 10 to 30 μg were already sufficient to cover the P2X7 receptor on all microglia and could be detected up to 21 days after injection (Additional file 1: Figs. S7, S8).

**Fig. 5:**
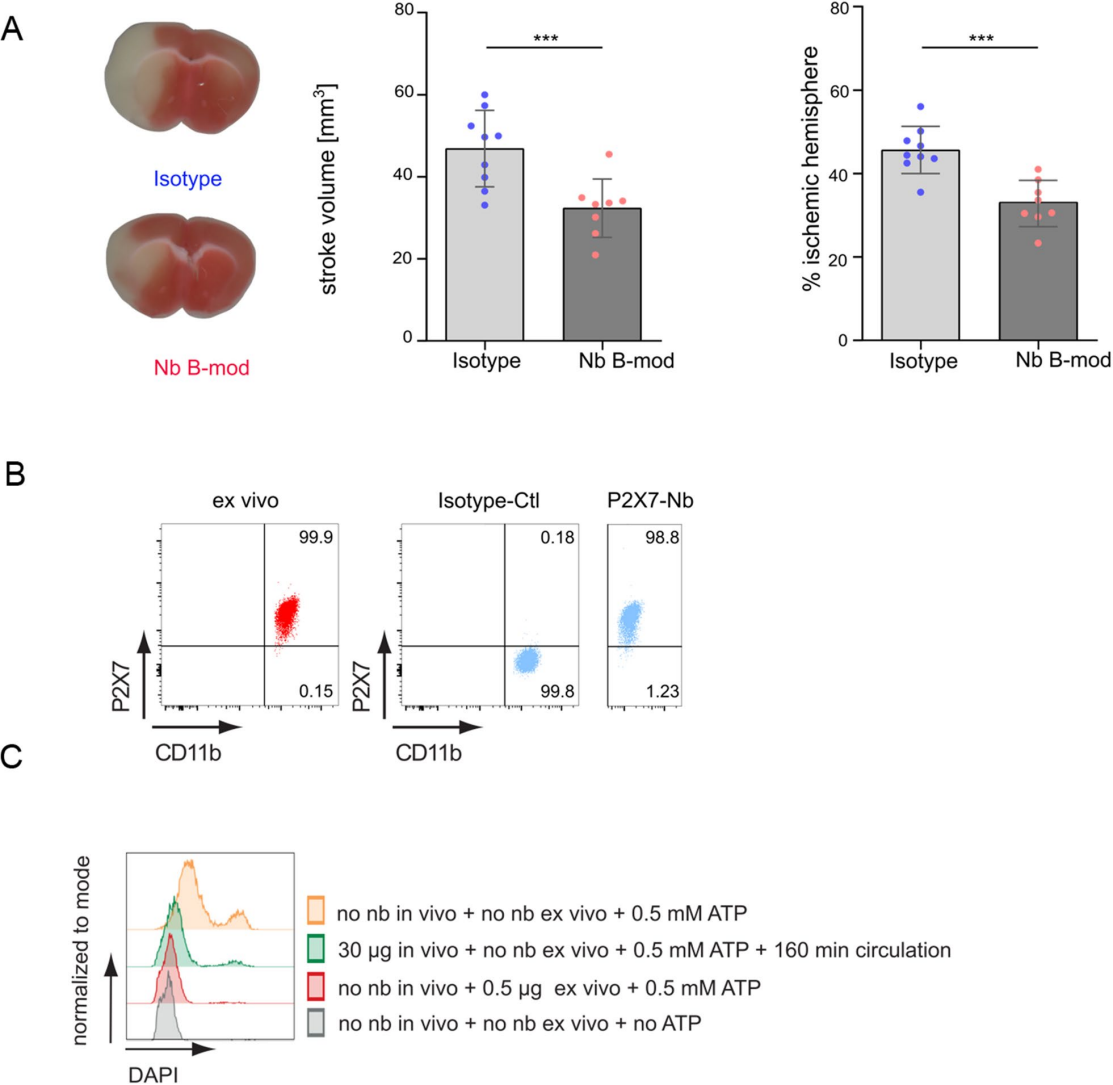
Icv injection of P2X7 nbs significantly reduced stroke size. P2X7-blocking nbs (30 μg) were injected intracerebroventricularly directly before tMCAO surgery of C57BL6J mice. Twenty-four hours after tMCAO, the mice were sacrificed. The mice treated with P2X7-blocking nbs showed significantly smaller ischemic volumes and a significantly smaller % of parenchyma loss in the ischemic hemisphere than the mice treated with a control isotype nbs. **A** Flow cytometry of brain resident microglia showed full coverage by P2X7-specific nbs compared to the controls (gating strategy Additional file 1: Fig. S2). Statistical significance was determined by Student’s t test. ***p < 0.001 Data are presented as the median ± SD of 8 P2X7-specific nb-injected mice and 9 isotype control-injected mice. **B** Representative TTC staining and representative overlays of stroke volume at 24 h following tMCAO. **C** 160 min after icv injection or ex vivo administration of 30 μg of P2X7-specific nbs, isolated microglia showed substantially lower DAPI uptake. Microglia in the absence of nbs and ATP did not show any DAPI uptake

Following our dose response curve we injected 30 μg of this modified P2X7-specific nb B-mod (*n* = 8) or an isotype control nb (*n* = 9) intracerebroventricularly into wildtype mice. Twenty-four hours after tMCAO, we analyzed the stroke size and found that the P2X7-specific nbs resulted in significantly decreased stroke sizes (26.16 mm^3^ ± 10.29 mm^3^ compared to isotype 42.02 mm^3^ ± 8.49 mm^3^; ***p < 0.01; Fig. 5A). This effect was also reflected by the loss of viable tissue (22.78% ± 8.84% compared to isotype 38.00% ± 8.32%; ***p < 0.01; Fig. 5A). Flow cytometry of brain resident microglia of these mice showed a strong signal of the P2X7-specific nbs (Fig. 5B). This signal could not be further increased by ex vivo addition of the P2X7-specific nbs. Functional P2X7 activation after icv injection of nbs was tested by analysis of ATP-induced DAPI uptake (Fig. 5C). Then, 160 min after icv injection of the P2X7-specific nbs, microglia were isolated from the brain and activated by ATP. Microglia exposed to the P2X7-specific nbs in vivo showed substantially lower DAPI uptake. After in vivo icv injection of 30 μg and administration of 0.5 mM ATP 61.9% of the microglia were protected from P2X7 activation and accordingly from DAPI-uptake. As a positive control DAPI-uptake in the absence of nbs and the presence of 0.5 mM ATP was 89.5%. The negative control without any nbs or ATP resulted in a DAPI-uptake of 1.4% (Table 2).

**Table 2:** DAPI-uptake of microglia after icv injection of P2X7 specific nb-B mod

	DAPI negative cells (in %)	DAPI positive cells (in %)
*no nb *in vivo + *no nb *ex vivo + *no ATP*	98.6	1.4
*no nb *in vivo + *0.5 μg *ex vivo + *0.5 mM ATP*	83.3	16.7
*30 μg *in vivo + *no nb *ex vivo + *0.5 mM ATP* + *160 min circulation*	61.9	38.1
*no nb *in vivo + *no nb *ex vivo + *0.5 mM ATP*	10.5	89.5

Functional analysis of P2X7 activation in microglia after icv injection of nbs was tested by checking the ATP-induced DAPI-uptake (Fig. 5C)

## Discussion

Here, we show that eATP is present early after cerebral ischemia and that blocking the ATP receptor P2X7 with specific nbs diminishes the tissue damage caused by ischemia. However, the nbs need to be injected intracerebroventricularly to bypass the BBB and reach the P2X7 receptor on brain resident cells.

Mounting evidence indicates that stroke triggers a sterile inflammatory response. The injured tissue releases a myriad of molecules that can activate the surrounding or infiltrating immune cells. Potent activators of local immune responses are danger-associated molecular patterns (DAMPs). Some of these endogenous danger signals can induce activation of the inflammasome and the secretion of proinflammatory cytokines by innate immune cells [4, 38]. Using transgenic mice that express luciferase on the outer layer of the cell membrane, we showed that similar to traumatic brain injury [39], eATP is released very early during ischemic tissue damage. In addition, the signal is sustained over 24 h, clearly indicating an ongoing release of eATP in the ischemic tissue. Therefore, eATP and its cognate receptors likely play an important role in the initiation of the inflammatory reaction following stroke. eATP activates purinergic receptors. While the microglial P2Y12 receptor is important for microglial neuron interactions, the proinflammatory response by microglia is likely triggered by P2X7, which is highly expressed by microglia [40–42].

P2X7 is expressed in the brain mainly on glial cells. Expression data from the Allen Brain Atlas for mice [18] and humans [19] show that the P2X7 receptor is highly expressed by microglia in humans and rodents (Table 1). In contrast, astrocytes show low levels of P2X7 RNA. Species-specific differences in P2X7 expression can be found for oligodendrocytes, where P2X7 is highly expressed by human oligodendrocytes but not by murine oligodendrocytes. Therefore, it is likely that in rodents, the main effect of blocking P2X7 is mediated through microglial cells.

After ischemic stroke, the expression of P2X7 is increased on microglia [43, 44] and can induce cell death in ischemic microglia [15]. This increase in P2X7 expression is not found in astrocytes after ischemic stroke [17]. We and others have shown that experimental stroke in P2X7-/- mice results in smaller infarcts and that blockade of P2X7 with BBG reduces cerebral ischemic damage [20, 45]. In addition, the inhibition of the NLRP3 inflammasome decreased the amount of damage after cerebral ischemia, but there was no additional benefit if P2X7 was also blocked [20]. These data are still controversial [12]. Yanagisawa and colleagues observed an exacerbation of ischemic brain damage when P2X7 was blocked. Similar findings were also reported by Kang et al. [46], who observed an effect on ciliary neurotrophic factor (CNTF) production but no effect on lesion size. One explanation for these discrepancies is the use of BBG. Small molecule inhibitors are often semispecific and toxic. In particular, BBG is not specific for P2X7 [47] and is known to have dose-dependent off-target effects. Therefore, we used nbs that we had recently developed and are currently in the process of being patented (see MM; WO/2013/178783) [10]. We not only generated several different families of murine P2X7-specific nbs but also different human P2X7-specific nbs. Nbs, recombinant single domain antibodies derived from camelid heavy chain antibodies, are a promising new technology platform. The first nb-based reagents developed by Ablynx-Sanofi have entered clinical trials and have achieved FDA approval (targeting TNF-α, von Willebrand factor, receptor activator of nuclear factor κB [RANK]-ligand, and IL-6 receptor [48, 49]).

The BBB is a major obstacle for the treatment of brain disease with biologicals. Under healthy conditions, the BBB is only permeable for lipophilic molecules of up to 400 kDa [50]. In addition, the delivery of conventional antibodies to the brain is further hampered by Fc receptor-mediated efflux to the blood [51]. Therefore, nbs lacking an Fc part may reach targets behind the BBB. However, under nonpathological conditions, monovalent nbs do not attain sufficient concentrations for in vivo brain imaging [52] or therapeutic purposes [53]. In stroke, biphasic BBB breakdown is caused by activated matrix metalloproteinase (MMP)-2, MMP-3 and MMP-9 [54, 55]. The breakdown of the BBB is initially reversible but is further increased with the release of MMP-3 and MMP-9 [56]. These findings suggest that antibodies or nbs would have easier access to the brain in ischemic stroke. However, as we can show here, only a minor portion of the intravenously injected nbs reached the brain. While macrophages from the bloodstream were quickly covered with intravenously injected nbs, when they reached the brain, microglia did not carry any nbs, and their function was unimpaired (Fig. 4). These findings are similar to observations in antibodies crossing the BBB, where a direct shuttle system such as the transferrin receptor is usually needed to enter the brain [57]. Since this problem prevents noninvasive iv administration of the nbs, it is necessary to find strategies to facilitate the transport of nbs across the BBB. For this study, we chose to directly inject our nbs into the ventricular system of the brain, which is difficult in the mouse system because of the small volume that can be injected. We were able to modify our nbs so they could be highly concentrated without aggregating (Additional file 1: Fig. S1). In humans, nb delivery would be less of a problem since it could be accomplished by lumbar puncture and injection into the cerebral spinal fluid (CSF). Direct injection in the CSF of therapeutics is already used for other neurological diseases, such as neuronal ceroid lipofuscinosis [58]. Other promising possibilities for nb delivery to the CNS include the fusion of nbs to ligands of brain-endothelial receptors such as ApoE-LDL-receptor or to nbs directed against cell transcytosis receptors on cerebral endothelial cells [59–61].

In stroke, microglia are the first immune cells to respond, while macrophages enter the brain at later stages [32]. Therefore, it is not surprising that there was no difference in ischemic lesion size after iv nb injection, where the nbs could not pass the BBB. In contrast, after an icv injection of P2X7-specific nbs, we could reach up to 95% of the microglia. This level of P2X7R blockade was sufficient to inhibit microglial activation and improve the outcome. Our study shows that inhibition of signaling by eATP is only effective if it is done early and reaches locally expressed P2X7 in the brain.

### Limitations

Our study was a proof-of-concept study, which was not designed to simulate the clinical setting. Further studies are needed to determine whether P2X7-specific nbs improve outcomes after stroke, how they influence long-term outcomes, and if they are similarly effective in female, comorbid and old mice. Our results will have to be reproduced in other laboratories and other model systems before translation.

### Conclusion

Here, we demonstrate the importance of locally produced eATP for the damage in ischemic stroke and the potential of intracerebroventricularly injected P2X7 nbs to reduce this damage.

## Supplementary Information

**Additional file 1: Figure S1.** The nbs used and their staining procedure. For this study, different nb constructs were used. The 13A7 nb (P2X7-specific nb; see patent WO/2013/178783; [10]) was fused to the hinge, CH2, and CH3 domains of mouse IgG2c, resulting in a heavy chain format (nb A), whereas 1c81 (P2X7-specific nb; see patent WO/2013/178783 [10]) was dimerized and fused to the albumin-specific nb Alb8 (nb B). To prevent aggregation at high concentrations, we modified nb B (nb B-mod). For recognition of these nbs in FACS, we used the following staining protocols: After binding of nb A to P2X7, cells were stained with a biotinylated anti-mouse IgG2c-fused antibody followed by streptavidin BV421 conjugation. After binding of nb B-mod, cells were stained with an anti-Alb8-nb fused to the mouse IgG1 heavy chain backbone, followed by an anti-mouse IgG1 antibody conjugated with BV421. **Figure S2.** Schematic representation of icv surgery. The cranial burr hole was drilled 1.1 mm lateral and 0.5 mm posterior to bregma. Nbs were injected 2.3 mm deep directly into the left ventricular system. As a proof-of-concept, 2 μl of 5% Evans blue was injected into the ventricular system. Two hours after injection, Evans blue was distributed equally in the whole ventricular system. **Figure S3.** Gating strategy for brain resident microglia. Flow cytometry of brain cells. Three minutes before euthanasia, a CD45-fluorochrome-conjugated antibody was injected intravenously to separate intravascular from intraparenchymal cells. Brain resident microglia were identified as CD45^intermed^ CD11b^high^ cells, which were not labeled by the intravenously injected CD45-fluorochrome conjugated antibody. **Figure S4.** In vitro calibration of brain homogenates from pmeLUC mice. The panel shows the in vitro calibration of brain homogenates from pmeLUC mice, showing the luminescence response to the addition of exogenous ATP and the obliteration of luminescence in the presence of the ATP-hydrolyzing enzyme apyrase. With all the caveats due to the in vitro setting, this calibration suggests that the eATP concentration in the stroked brain may reach hundreds of micromoles/L. **Figure S5.** Negative control. In GFP-negative littermates, no P2X7 signal was found. **Figure S6.** High doses of P2X7-blocking nbs are necessary to cross the BBB efficiently. C57BL6J mice received different amounts of nb A intravenously. Four hours after iv injection mice were sacrificed and nbs bound to microglia were labeled by FACS (See MM). Full coverage of P2X7 was reached with 3200 μg, where 1000 μg and lower concentrations achieved less than 60% occupancy of microglial P2X7. These FACS data are representative images of two independent cohorts of 5 mice each. **Figure S7.** Low amounts of icv injectedP2X7-blocking nb B-mod showed high P2X7 occupancy on microglia. C57BL6J mice received different amounts of P2X7-specific nbs intracerebroventricularly. 18 h after icv injection mice were sacrificed and nbs bound to microglia were labeled by FACS (See MM). Low amounts of P2X7 blocking nb B-mod were needed to show almost full occupancy of microglial P2X7 receptor. These FACS data are representative images of two independent cohorts of 5 mice each. **Figure S8.** Icv injection of P2X7-blocking nb B-mod resulted in a long time P2X7 occupancy on microglia. C57BL6J mice received 30 μg P2X7-specific nb-B mod intracerebroventricularly. Mice were sacrificed at different time points after icv injection and nbs bound to microglia were labeled by FACS (See MM). After 2.5 h microglial P2X7 showed nearly complete coverage by P2X7 nbs. This high occupancy started to decrease 14 days after the icv injection, but still nearly 40% of microglial P2X7 was occupied after 21 days post icv injection. These FACS data are representative images of two independent cohorts of 6 mice each.

## Data Availability

The datasets used and analyzed during the current study are available from the corresponding author on reasonable request.

## Abbreviations

nbs: Nanobodies
wt: Wild-type
tg: Transgenic
tMCAO: Temporary middle cerebral artery occlusion
iv: Intravenous
ip: Intraperitoneal
icv: Intracerebroventricular
DAPI: 4,6-Diamidino-2-phenylindole
IL: Interleukin
(e)ATP: (Extracellular) adenosine triphosphate
FACS: Fluorescence-activated cell sorting
h: Hours
NAD: Nicotinamide adenine dinucleotide
HSP: Heat shock protein
HMGB1: High-mobility group box 1 protein
NFκB: Nuclear factor kappa-light-chain-enhancer of activated B cells
NLRP3: Nod-like receptor protein 3
TLR: Toll-like receptors
TNF: Tumor necrosis factor
CNS: Central nervous system
BBG: Brilliant blue G
sdAbs: Single domain antibodies
CDR3: Complementarity determining region 3
(E)GFP: Enhanced green fluorescent protein
CH: Constant domain heavy chain
Ig: Immunoglobulin
SDS-PAGE: Sodium dodecyl sulfate–polyacrylamide gel electrophoresis
CCA: Common carotid artery
ECA: External carotid artery
MRI: Magnetic resonance imaging
TTC: Triphenyl tetrazolium chloride
PBS: Phosphate-buffered saline
CD: Cluster of differentiation
BV: Brilliant violet
FLICA: Fluorochrome-labeled inhibitors of caspases
ELISA: Enzyme-linked immunoassay
PFA: Paraformaldehyde
IbA1: Ionized calcium-binding adapter molecule 1
NeuN: Neuronal nuclear protein
BBB: Blood–brain barrier
MFI: Mean fluorescence intensity
DAMPs: Damage-associated molecular patterns
RNA: Ribonucleic acid
MFCNTF: Ciliary neuronotrophic factor
RANK: Receptor activator of nuclear factor k
MMP: Matrix metalloproteinases
CSF: Cerebral spinal fluid
ApoB: Apolipoprotein B
LDL: Low density lipoprotein receptor
