# Supplementary figures and images for: Blocking P2X7 by intracerebroventricular injection of P2X7-specific nanobodies reduces stroke lesions
Source: J Neuroinflammation. 2022 Oct 12;19:256. doi: 10.1186/s12974-022-02601-z (PMC9559872; doi:10.1186/s12974-022-02601-z)

Supplementary Figures

1


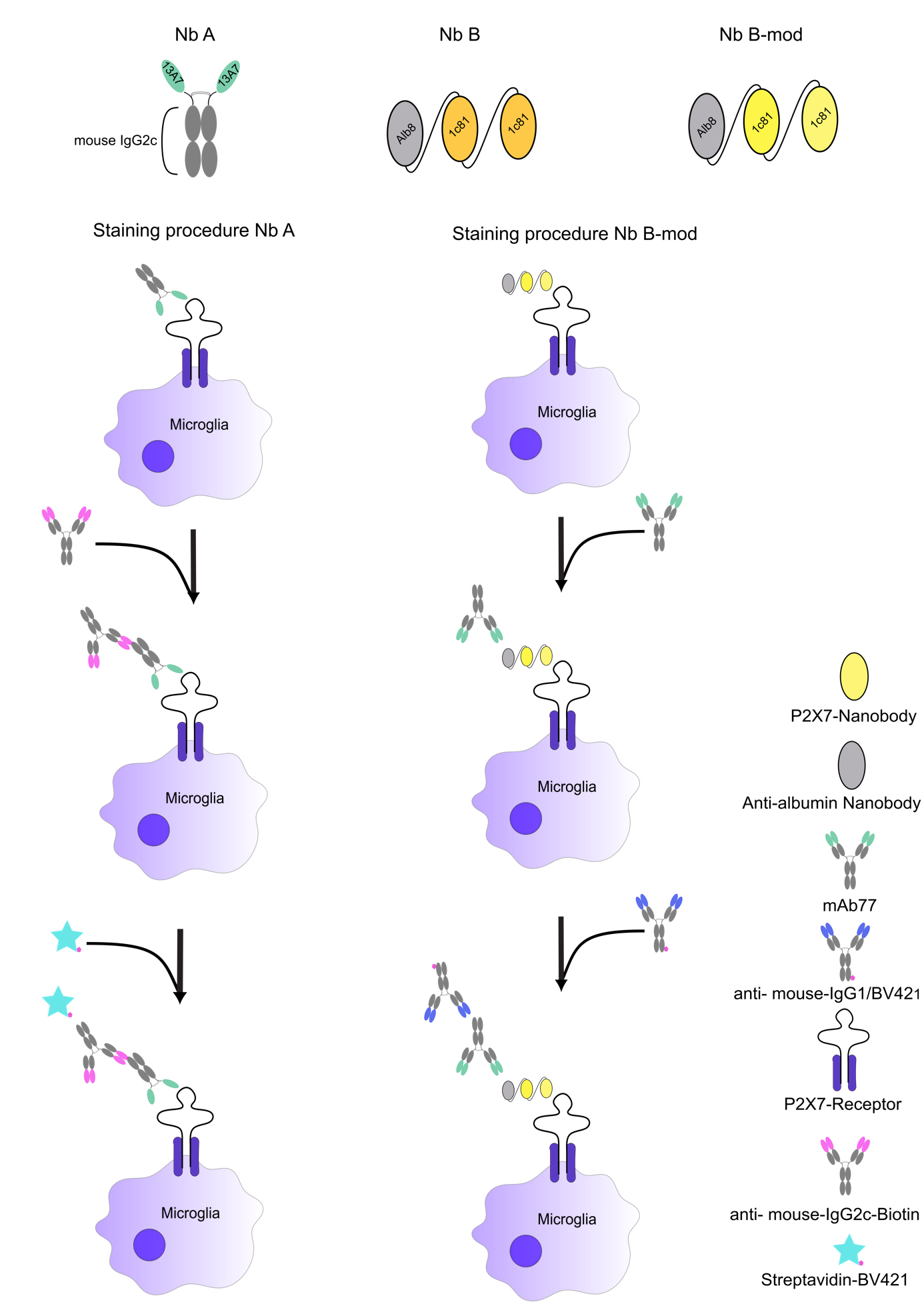


2


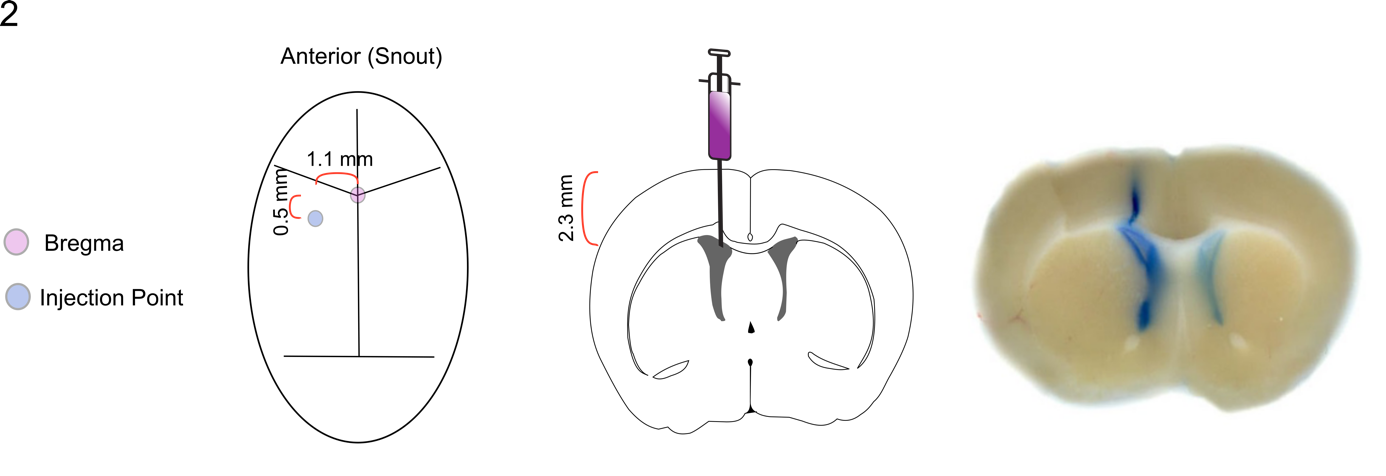


3


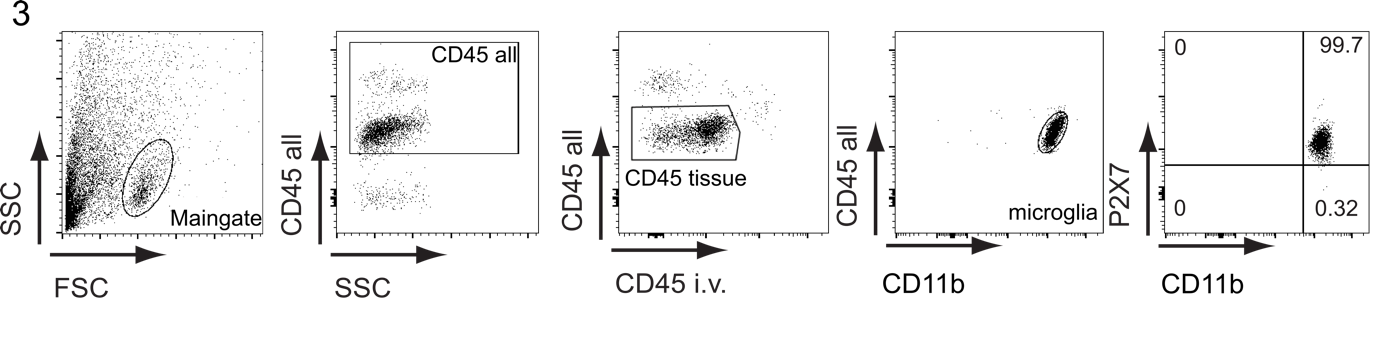


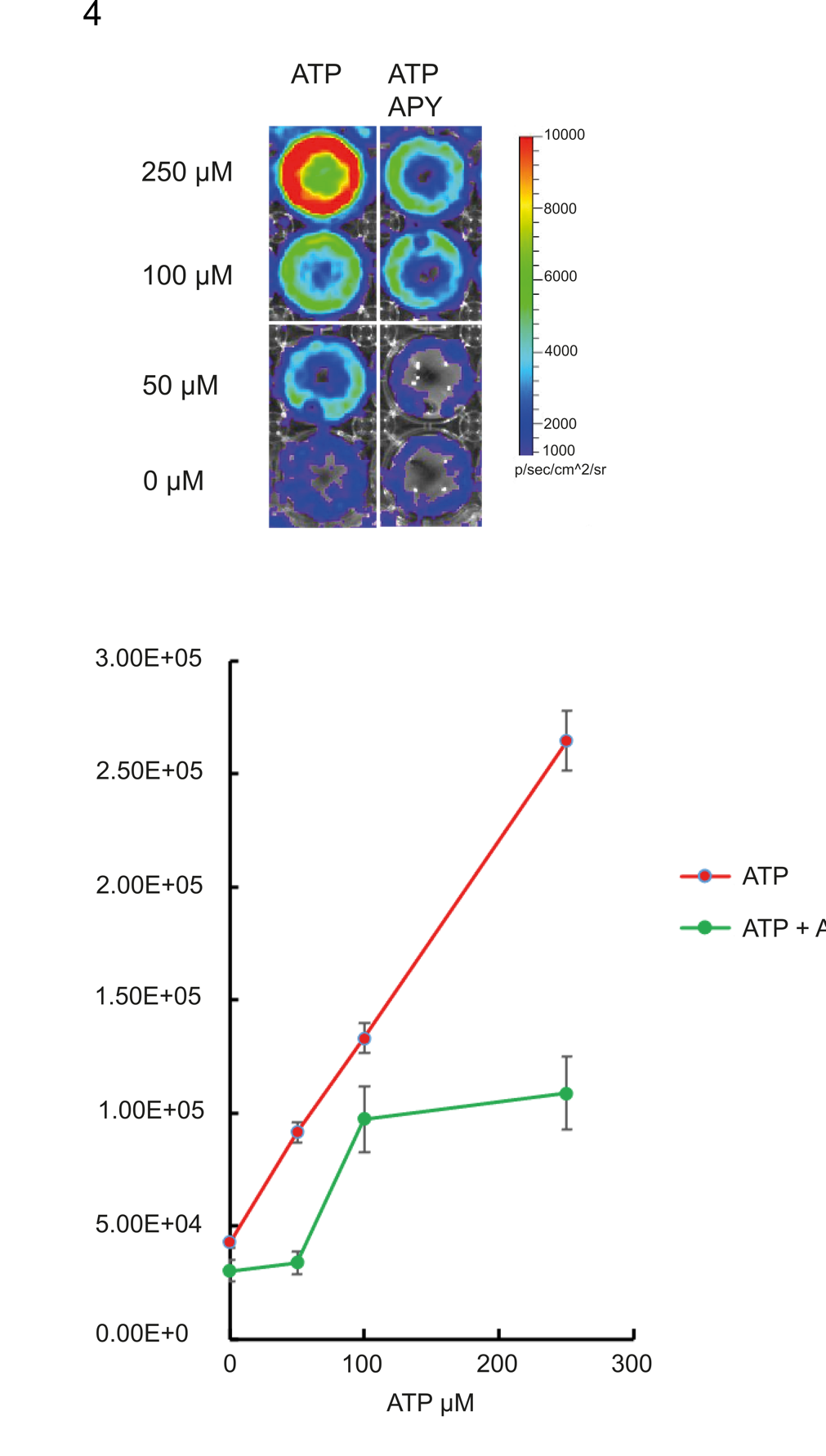


5


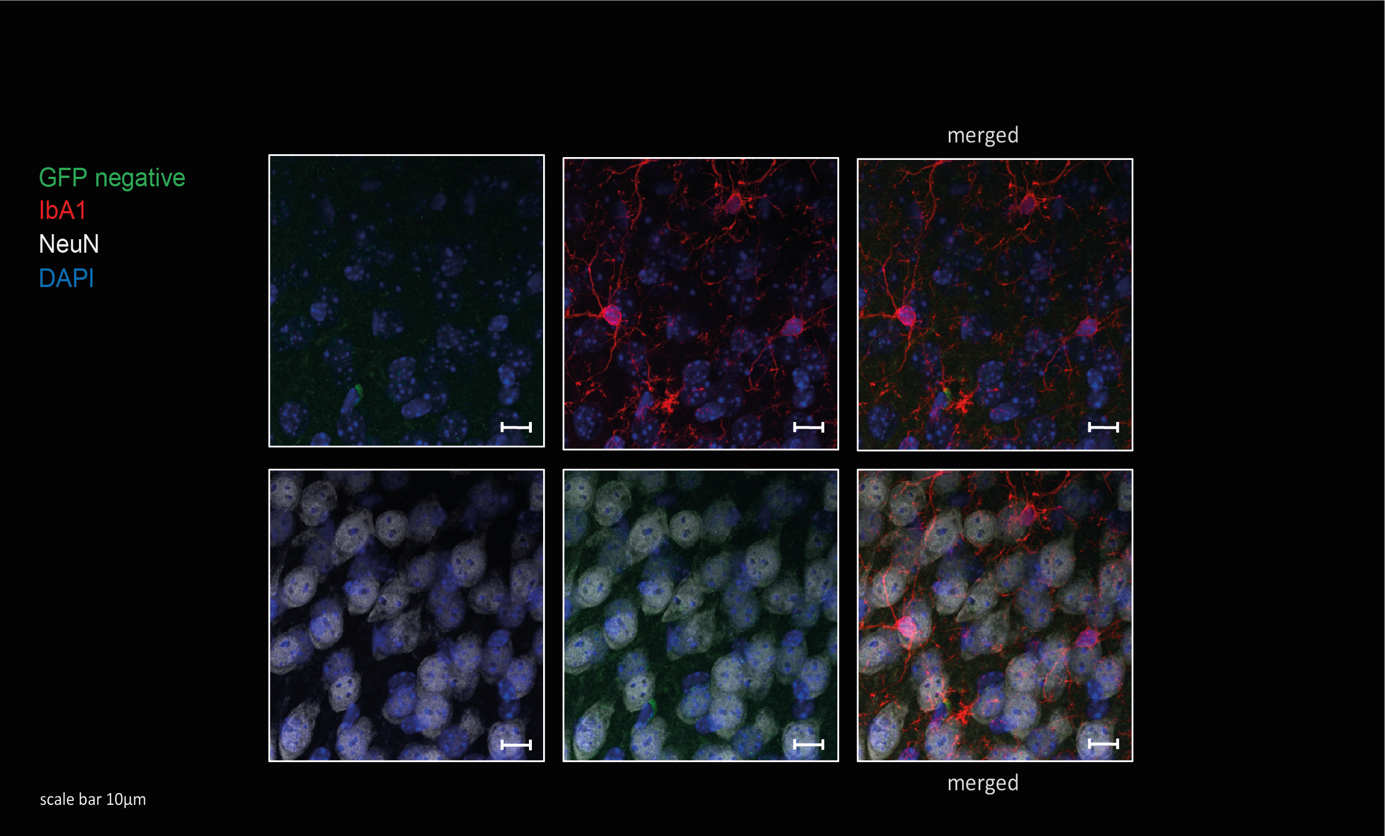


6


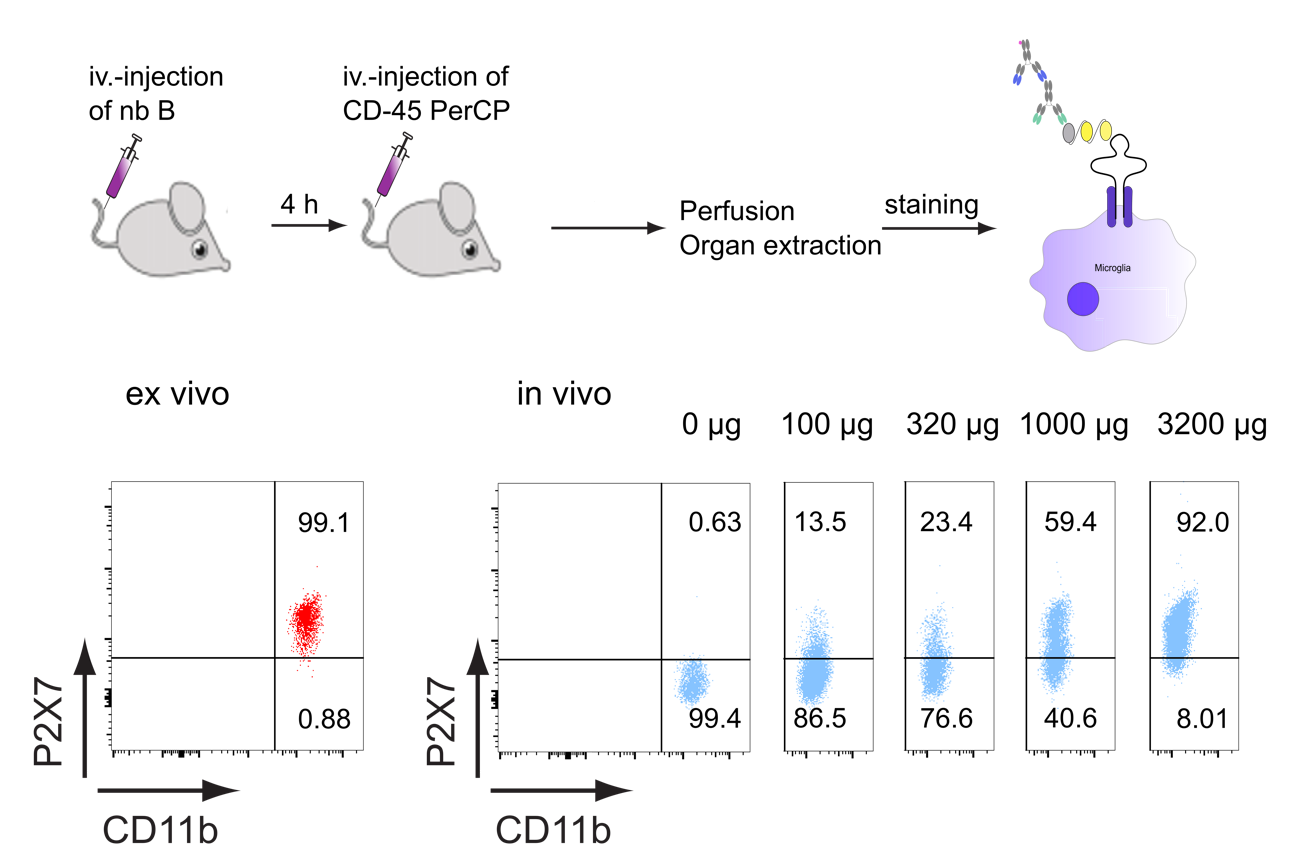


7


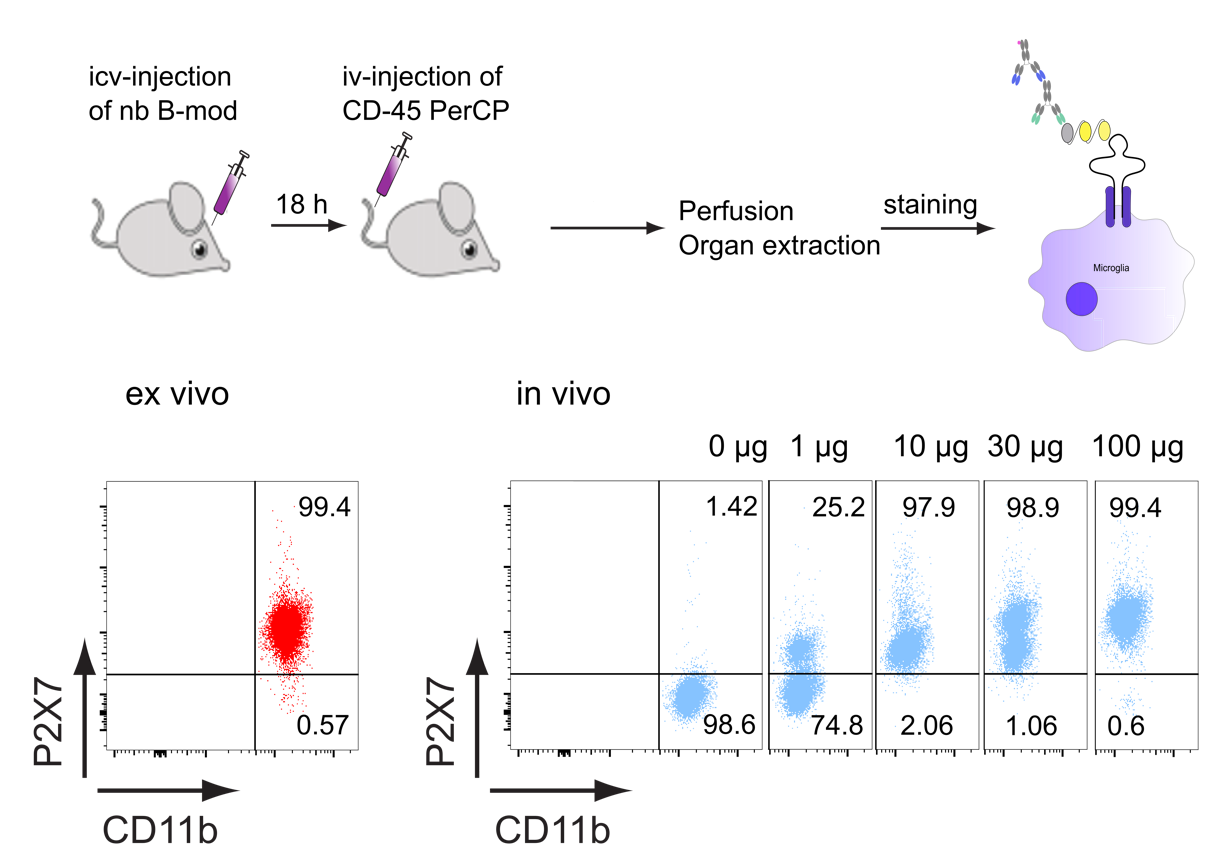


8


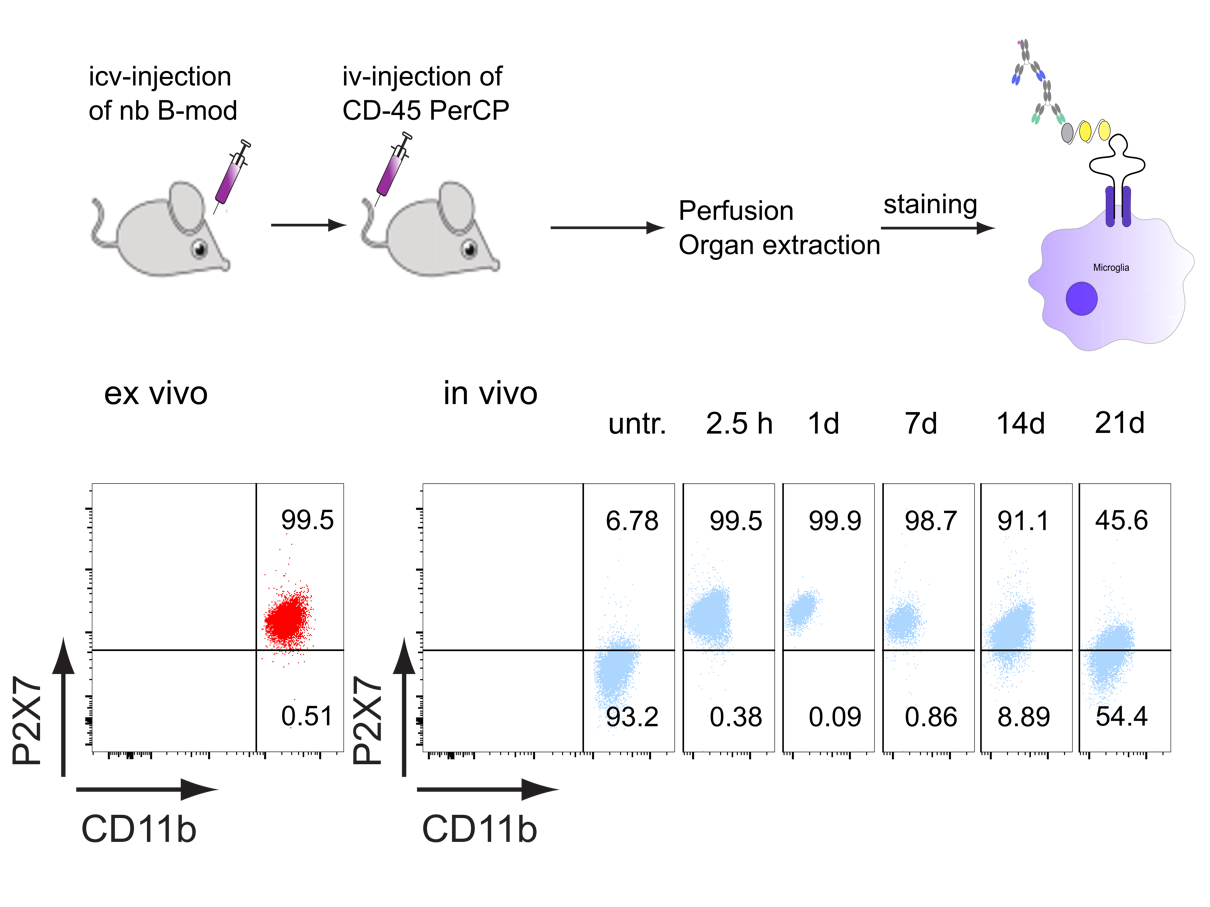

Supplement: Supplementary file 1 — Additional file 1: Figure S1. The nbs used and their staining procedure. For this study, different nb constructs were used. The 13A7 nb (P2X7-specific nb; see patent WO/2013/178783; [10]) was fused to the hinge, CH2, and CH3 domains of mouse IgG2c, resulting in a heavy chain format (nb A), whereas 1c81 (P2X7-specific nb; see patent WO/2013/178783 [10]) was dimerized and fused to the albumin-specific nb Alb8 (nb B). To prevent aggregation at high concentrations, we modified nb B (nb B-mod). For recognition of these nbs in FACS, we used the following staining protocols: After binding of nb A to P2X7, cells were stained with a biotinylated anti-mouse IgG2c-fused antibody followed by streptavidin BV421 conjugation. After binding of nb B-mod, cells were stained with an anti-Alb8-nb fused to the mouse IgG1 heavy chain backbone, followed by an anti-mouse IgG1 antibody conjugated with BV421. Figure S2. Schematic representation of icv surgery. The cranial burr hole was drilled 1.1 mm lateral and 0.5 mm posterior to bregma. Nbs were injected 2.3 mm deep directly into the left ventricular system. As a proof-of-concept, 2 μl of 5% Evans blue was injected into the ventricular system. Two hours after injection, Evans blue was distributed equally in the whole ventricular system. Figure S3. Gating strategy for brain resident microglia. Flow cytometry of brain cells. Three minutes before euthanasia, a CD45-fluorochrome-conjugated antibody was injected intravenously to separate intravascular from intraparenchymal cells. Brain resident microglia were identified as CD45intermed CD11bhigh cells, which were not labeled by the intravenously injected CD45-fluorochrome conjugated antibody. Figure S4. In vitro calibration of brain homogenates from pmeLUC mice. The panel shows the in vitro calibration of brain homogenates from pmeLUC mice, showing the luminescence response to the addition of exogenous ATP and the obliteration of luminescence in the presence of the ATP-hydrolyzing enzyme apyra [file 12974_2022_2601_MOESM1_ESM.docx]
